# Primary squamous cell carcinoma of the pancreas: a case report and review of the literature

**DOI:** 10.1186/1752-1947-6-295

**Published:** 2012-09-13

**Authors:** Ravi Kodavatiganti, Fiona Campbell, Ahmed Hashmi, Simon W Gollins

**Affiliations:** 1 North Wales Cancer Treatment Centre, Glan Clwyd Hospital, Sarn Lane, Denbighshire, LL18 5UJ, UK; 2 Department of Histopathology, The Royal Liverpool and Broadgreen University Hospitals NHS trust, Prescot street, Liverpool, L7 8XP, UK

## Abstract

**Introduction:**

Primary squamous cell carcinoma of the pancreas is a rare tumor with poor prognosis and is found in the literature only as case reports. The optimal management course remains poorly defined. We present a case of primary basaloid squamous cell carcinoma of the pancreas metastatic to the liver, which was treated with surgery and systemic chemotherapy. Our patient survived for 15 months: the longest survival reported in the literature to date.

**Case presentation:**

A 70-year-old Caucasian man presented to hospital with a three-month history of weight loss, pruritus and icterus. Imaging studies confirmed the presence of an operable mass lesion in the head of the pancreas. Following a pancreaticoduodenectomy, histology results led us to make a diagnosis of squamous cell carcinoma. Postoperative restaging showed multiple metastases in the liver. He underwent palliative systemic chemotherapy with cisplatin and 5-fluorouracil achieving partial response and an excellent quality of life. He then went on to start second-line chemotherapy, but unfortunately died of sepsis soon thereafter.

**Conclusions:**

This case report emphasizes that achievement of a worthwhile objective and symptomatic palliative response is possible using platinum-based chemotherapy in squamous cell carcinoma of the pancreas.

## Introduction

Squamous cell carcinoma of the pancreas is rare. Squamous cells are not present in the normal pancreas and hence the pathogenesis of this carcinoma remains uncertain. Even though squamous cell carcinomas arising in other parts of the body are considered to be radiosensitive and chemosensitive, and sometimes have better outcomes, the prognosis of squamous cell carcinoma in the pancreas remains poor, as for other pancreatic carcinomas.

## Case presentation

A 70-year-old Caucasian man who was a non-smoker presented to our facility with a three-month history of 12kg weight loss and recent generalized itching and jaundice. Serum biochemistry and ultrasound of the abdomen confirmed obstructive jaundice due to a mass in the head of the pancreas. The lesion was 4.6×4.1cm in size with no evidence of metastatic disease on staging computed tomography (CT) scan. He underwent endoscopic retrograde cholangiopancreatography (ERCP) with a 5cm 10F plastic stent inserted into the common bile duct followed by pylorus-sparing pancreaticoduodenectomy two months after diagnosis and recovered uneventfully.

**Figure 1:**
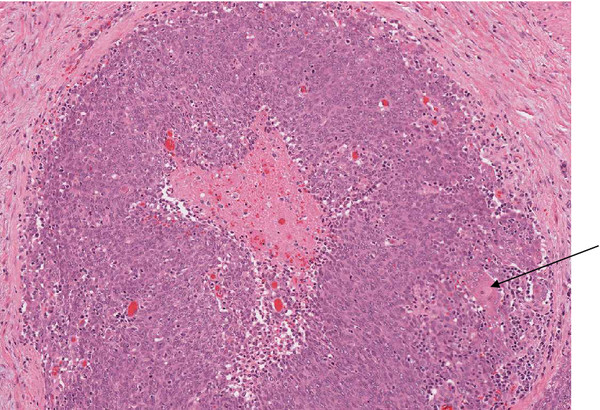
Low power image of the carcinoma with central necrosis and focal squamous differentiation at right edge (arrow).

**Figure 2:**
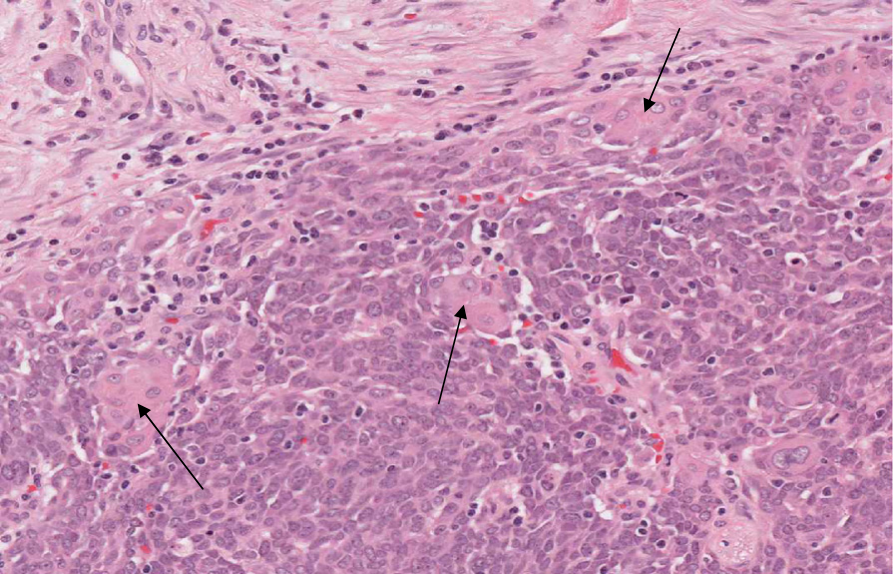
Higher power image with desmoplastic stroma at top and squamous pearls amongst the carcinoma cells (arrows).

Macroscopically, there was a 5.5cm diameter, lobulated tumor within the head of the pancreas extending to the superior mesenteric vessel margin. This entire tumor was sampled for histological examination. Microscopically, the tumor was composed of large nests of basaloid cells with areas of central necrosis (Figure 1) and scattered small foci of squamoid differentiation (Figure 2). There was no evidence of acinar or glandular differentiation morphologically. There was extensive intravenous and intralymphatic invasion, together with foci of perineural invasion. The tumor invaded into the duodenum, around the base of the ampulla, into the pancreatic duct and widely into peripancreatic fat. Tumor involvement of the superior mesenteric vessel margin was confirmed microscopically. Metastatic tumor (with similar morphology) was present in six of 43 sampled lymph nodes.

The morphological appearances of the tumor were considered to be those of a poorly differentiated (basaloid) squamous cell carcinoma, which was confirmed with diffuse strong immunostaining with cytokeratin 5/6 and p63. Adenosquamous carcinoma and pancreatoblastoma were considered in the differential diagnosis. Adenosquamous carcinoma is a rare variant of pancreatic ductal adenocarcinoma, showing both glandular differentiation and squamous differentiation. However, no glandular differentiation was seen in our tumor, despite extensive sampling. Pancreatoblastoma occurs in childhood, but rare cases have been described in adults. By definition, it shows acinar differentiation and squamoid nests. However, our tumor showed no acinar growth pattern and no immunostaining with trypsin or α-fetoprotein. There were only occasional neuroendocrine cells (immunopositive for synaptophysin and CD56) within the tumor, ruling out a neuroendocrine carcinoma.

Squamous cell carcinoma of the pancreas is extremely rare and, therefore, the possibility was raised that this could be secondary involvement of the pancreas. A repeat staging CT scan was performed postoperatively and revealed possible metastatic spread to the liver, but did not identify any other tumor sites. He underwent an [18F]-2-fluoro-2-deoxy-d-glucose (FDG) positron emission tomography-computed tomography (PET-CT) scan one month postoperatively, which was reported to show FDG avid lesions in both lobes of the liver and a solitary FDG avid lymph node anterior to the renal vein. Again, no other occult potential primary site was visible. Two months after surgery, he started systemic palliative chemotherapy with intravenous cisplatin at 80mg/m^2^ given on day one and 5-fluorouracil 4000mg/m^2^ as continuous intravenous infusion over four days (days one to five). On completion of eight three-weekly cycles, restaging CT scan demonstrated what amounted to a partial response in his liver metastases by Response Evaluation Criteria In Solid Tumors (RECIST) scoring. Following this he was then placed on follow-up, achieving an excellent quality of life.

He remained active but developed epigastric pain six months later. A restaging CT scan showed progression in the previously noted metastatic liver disease and a 3.3cm mass in the retroperitoneum, suggestive of local recurrence. As he remained active with performance status of 1 on the WHO scale, he commenced second-line chemotherapy with docetaxel a month later. Four days after the first cycle of docetaxel had been administered he was admitted to hospital with sepsis. Blood cultures obtained on admission grew *Klebsiella pneumoniae* and *Clostridium perfringens* sensitive to ciprofloxacin, tazocin, gentamicin and metronidazole. Despite treatment with tazocin and gentamicin he died of sepsis within 48 hours of admission.

## Discussion

Primary squamous cell carcinoma of the pancreas is an extremely rare entity with an incidence of 0.5% to 2% of all exocrine pancreatic neoplasms [1]. Cases are described in the literature only as case reports [2-11]. In view of its rarity, squamous cell carcinoma (SCC) in the pancreas is presumed to be metastatic from another primary site, until proven otherwise [1,8]. However, it should be noted that metastases to the pancreas are also rare. Metastatic spread of primary lung SCC to the pancreas is noted to be common in both surgical and autopsy series with pancreatic involvement usually occurring as part of widespread disease [1]. A case of an isolated metastasis from asymptomatic occult esophageal squamous cell cancer to the pancreas has also been reported to masquerade as primary pancreatic cancer [12].

**Figure 3:**
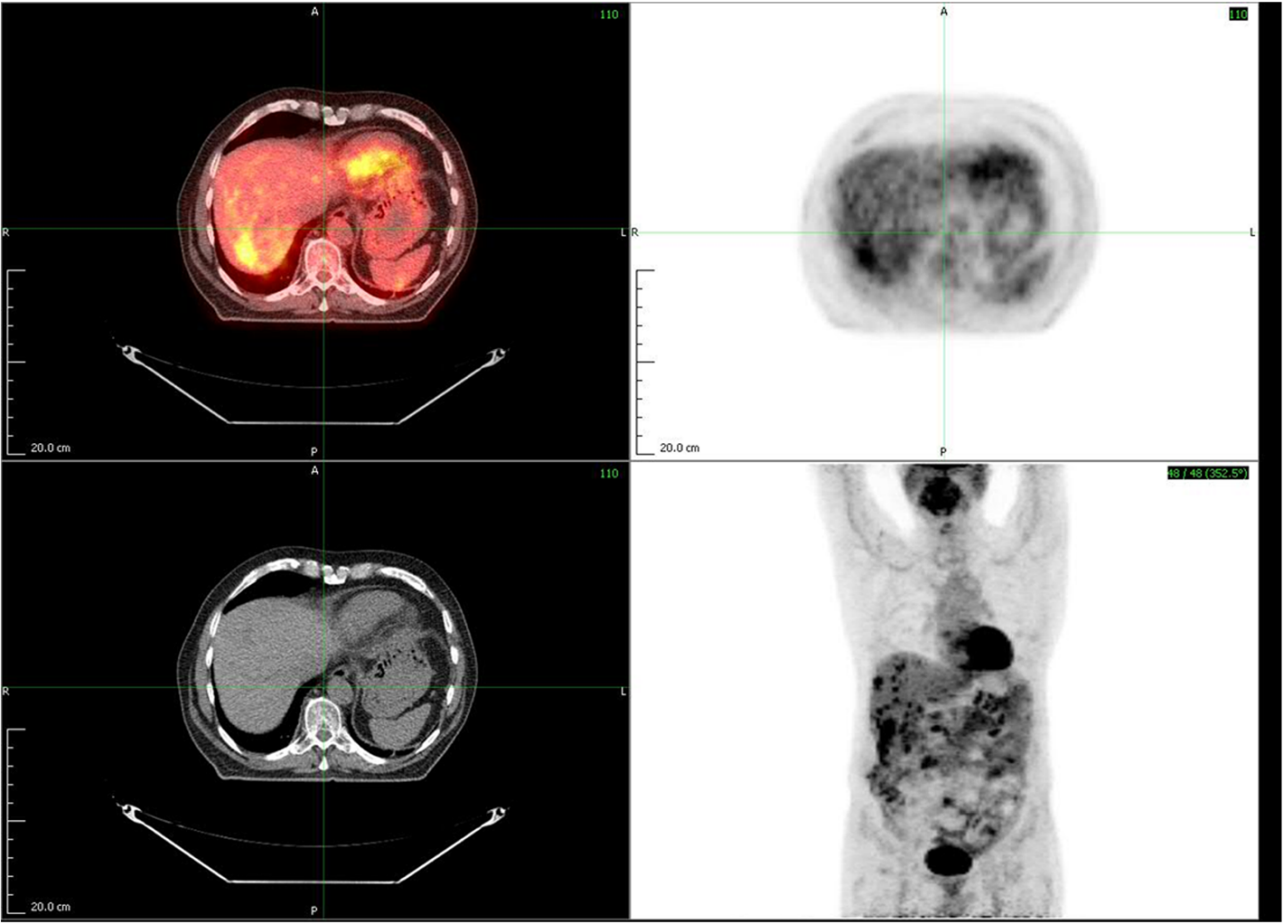
Positron emission tomography-computed tomography scan showing multiple liver metastasis immediately post-pancreaticoduodenectomy and prior to first-line palliative chemotherapy.

**Figure 4:**
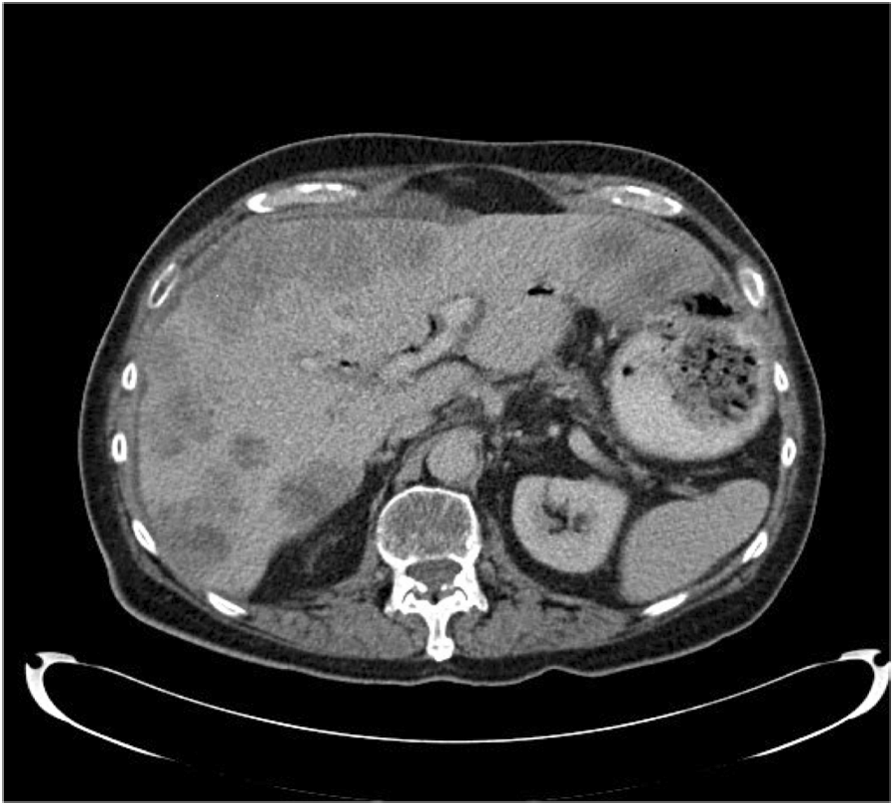
Computed tomography scan showing multiple liver metastases prior to second-line palliative chemotherapy.

Our patient was presumed to have operable primary pancreatic cancer. The surprise histological finding of squamous cell carcinoma raised the possibility that this may have been a metastatic deposit and a PET-CT scan was thus performed to attempt to localize a possible occult primary. However, the PET-CT scan showed only multiple liver metastases and no other candidate primary site (Figures 3 and 4). Historically, most of the reported cases used CT scans of head, neck and chest, otorhinolaryngological examination and endoscopic examination of the gastrointestinal tract to search for the primary. To the best of our knowledge, this is the only reported case to have used a PET-CT scan to search for the primary in this context.

We undertook a MEDLINE database search to identify cases of pure squamous cell carcinoma of the pancreas reported in the English literature and their management and outcomes using the following key words: carcinoma, squamous cell, pancreas, exocrine, pancreatic neoplasms. Brown *et al*. in 2005 identified 36 autopsy/registry cases of pure squamous cell carcinoma of the pancreas and 25 cases diagnosed based on ante-mortem histology results [10]. We have identified (Table 1) an additional 14 cases diagnosed ante-mortem since 2005 and a further case diagnosed ante-mortem in 2000 by Fonseca *et al*. [11], which was not included in the excellent review by Brown *et al*. [10]. Including the present case, a total of 40 cases of pure squamous cell carcinoma of the pancreas, diagnosed based on histology findings, have been reported in the English literature.

**Table 1:** Reported cases of squamous cell carcinoma of the pancreas in the literature

**Author/reference**	**Year of publication**	**No. of patients reported**	**Age**	**Sex**	**Race**	**Presenting symptom(s)**	**Location**	**Size**	**Surgery**	**Outcome**	**Treatment**	**Time to death (months)**
Kensuke [2]	2011	1	67	F	Japanese	Anorexia, back pain	Tail	6cm	Distal panreatectomy, total gastrectomy, splenectomy.	LR at four months	Radiotherapy for LR	11 months from S
Mansfield *et al*. [3]	2010	8	64 (median)	6M+2F	NA	Jaundice (4/8), abdominal discomfort (3/8), back pain (1/8), early satiety (1/8)	NA	NA	1 (S), 1(S+R), 3 (S+R+C), 1 (C), 2 (P)	NA	NA	Median five months
Terada [4]	2010	1	69	F	Japanese	Abdominal pain, jaundice	Head	5cm	Pancreaticoduodenect- omy	Distant metastasis	Nil	Three months
Lai *et al*. [5]	2009	1	76	F	African-American	Epigastric/back pain, weight loss	Tail	5cm	Cytological diagnosis, no histology	Liver metastasis at diagnosis	Not known	Not known
Rana *et al*. [6]	2009	1	50	M	NA	Abdominal pain, jaundice, weight loss	Head	NA	No surgery, cytological diagnosis, no histology	Peritoneal deposits, ascites at diagnosis	Nil	Not known
Qiang‒pu *et al*. [7]	2008	1	55	M	Mandarin Chinese	Jaundice, weight loss	Head	4cm	Pancreaticoduodenect- omy and segmental liver resection	Liver metastasis at 10 months	Nil	10 months
Al-Shehri *et al*. [8]	2008	1	48	F	NA	Abdominal pain, jaundice, weight loss	Head	4.4cm	Palliative bypass surgery	Liver metastasis at diagnosis	Carboplatin+gemcitabine (two courses)	Five months OS from presentation
Anagnostopoulos *et al*. [9]	2006	1	72	M	NA	Painless jaundice	Head	6cm	Palliative bypass surgery		Nil	Four months
Brown *et al*. [10]	2005	1 (plus 24 case literature review)	As published in literature									
Fonseca *et al*. [11]	2000	1	52	M	NA	Melena, jaundice	Head	NA	No surgery, cytological diagnosis, confirmed histology at autopsy	Liver metastasis at diagnosis	Nil	712 days

*Abbreviations: S* Surgery, *R* Radiotherapy, *C* Chemotherapy, *P* Palliative care, *LR* Local relapse, *OS* Overall survival, *NA* Not available.

The normal pancreas is devoid of squamous cells and the origin of pancreatic SCC is uncertain. Various mechanisms postulated for evolution of pure squamous cell carcinoma include malignant transformation of squamous metaplasia, squamous metaplastic change in a pre-existing adenocarcinoma, and differentiation with malignant transformation of primitive multipotent cells [1]. Squamous metaplasia occurs in chronic pancreatitis and following pancreatic/biliary stents. However, our patient had no previous episodes of pancreatitis and the stent was only inserted pre-operatively.

Surgery still remains the corner stone in the management of this rare cancer as for the much more common ductal adenocarcinoma of the pancreas. Median survival for pancreatic SCC was noted to be seven months, with a range of six to 16 months, in the seven patients who underwent curative resection [10]. In our literature search we identified three patients whose overall survival was three, 10 and 11 months following curative resection [2,4,7]. We could not establish the overall survival of the fourth patient who had curative resection from the Mayo clinic series [3]. Median survival for those who did not have curative resection was three months (range one-quarter of a month to nine months) [3,10]. A total of 17 out of 39 patients had treatment for locally advanced, recurrent or metastatic squamous cell cancer of pancreas. The treatment included systemic chemotherapy and/or radiotherapy. Cisplatin was used in combinations with fluorouracil (three patients) [10,13], etoposide (one patient) [10] and vinblastine (one patient) [10]. Gemcitabine was used in combination with carboplatin (one patient) [8] and fluorouracil (one patient) [10]. The survival in these patients was poor except for one patient who recurred locally after surgery at four months. He received local radiotherapy and had an overall survival of 11 months.

Even though our patient relapsed early after surgery with multiple liver metastases, he appears to have benefited considerably from palliative systemic chemotherapy. He achieved an overall survival of 15 months from the surgery. He tolerated chemotherapy well and had a good quality of life during chemotherapy and for the majority of the seven months between completing chemotherapy and his presentation with symptomatic relapse. We elected to treat our patient with a platinum-based systemic chemotherapy regimen as it is active and used in various combinations for treatment of squamous cell carcinomas arising at different sites including lung, head and neck, cervix and skin.

## Conclusions

Pure primary squamous cell carcinoma of the pancreas is a rare carcinoma and, therefore, spread from other primary squamous cell cancers should always be considered and carefully excluded. Surgery remains the corner stone of treatment but unfortunately is not curative with the clinical course complicated by local and/or distant relapses. This current case report emphasizes that achievement of a worthwhile objective and symptomatic palliative response is possible using platinum-based chemotherapy in squamous cell carcinoma of the pancreas. The treatment of these rare cancers is challenging and may only be improved by centralized national registries.

## Consent

Written informed consent was obtained from the patient’s next of kin for publication of this case report and any accompanying images. A copy of the written consent is available for review by the Editor-in-Chief of this journal.

## Competing interests

The authors declare that they have no competing interests.

## Authors’ contributions

RK assessed our patient on presentation following surgery, arranged restaging investigations, planning of systemic chemotherapy, supervision of treatment and writing of the case report. FC reported the histopathology findings, provided the microphotographs and contributed to the writing of the case report. AH was involved in the literature search and writing up the case report. SWG was the supervising consultant in charge of patient management and helped in writing the manuscript. All authors read and approved the final manuscript.
